# DyCoNet: A Gephi Plugin for Community Detection in Dynamic Complex Networks

**DOI:** 10.1371/journal.pone.0101357

**Published:** 2014-07-07

**Authors:** Julie Kauffman, Aristotelis Kittas, Laura Bennett, Sophia Tsoka

**Affiliations:** 1 Department of Informatics, King's College London, Strand, London, United Kingdom; 2 Centre for Process Systems Engineering, Department of Chemical Engineering, University College London, Torrington Place, London, United Kingdom; Tel Aviv University, Israel

## Abstract

Community structure detection has proven to be important in revealing the underlying organisation of complex networks. While most current analyses focus on static networks, the detection of communities in dynamic data is both challenging and timely. An analysis and visualisation procedure for dynamic networks is presented here, which identifies communities and sub-communities that persist across multiple network snapshots. An existing method for community detection in dynamic networks is adapted, extended, and implemented. We demonstrate the applicability of this method to detect communities in networks where individuals tend not to change their community affiliation very frequently. When stability of communities cannot be assumed, we show that the sub-community model may be a better alternative. This is illustrated through test cases of social and biological networks. A plugin for Gephi, an open-source software program used for graph visualisation and manipulation, named “DyCoNet”, was created to execute the algorithm and is freely available from https://github.com/juliemkauffman/DyCoNet.

## Introduction

Community structure as a modular architecture is common in complex systems, where communities are defined as groups of nodes with dense intra-community edges and sparse inter-community connections [1], [2]. Generally, nodes in the same community have been found to share common properties or play similar roles in the organisation of the network [3], often corresponding to a functional unit in the system [4]. The detection of such communities, also known as *modules*, has proven important in the investigation of the underlying principles governing complex systems and has been a very active area of research over the past decade. Community structure has been considered primarily in the context of static networks, however in reality complex systems are not static; entities and their interactions can be created or equally, cease to exist, resulting in dynamic effects [5], [6]. It follows that a current challenge in community structure detection is the incorporation of temporal information into network modelling frameworks.

Community structure is a defining characteristic of networks across various disciplines [2]. An area in which such methodology has proven useful is the analysis of biological networks. In biological systems, groups of interacting proteins are underlying cellular processes [7], and therefore identifying these groups is crucial to understanding cellular function. Community structure detection methods have previously served to propose functionally coherent modules in static protein protein interaction (PPI) networks [8]–[10], however the introduction of dynamic effects in terms of spatial, temporal and environmental conditions can result in more accurate mechanistic models [11], [12].

In biological networks, dynamics can represent time steps of cellular processes within a specific organism or evolutionary time points. For example, it has been shown that yeast PPI networks have become more modular over evolutionary time [13]. Furthermore, modules in networks generated from time course gene expression data were found to be more informative than those detected from static PPI networks [14], [15]. These examples illustrate the potential for dynamic community detection methods to provide more informative insights into biological networks and emphasise the need for accurate and robust methodologies to facilitate novel applications.

Here, we provide a software implementation of a methodology for (i) identifying the stability of node-community membership and communities over time, and (ii) deriving sub-communities of nodes with highly variable community membership which follow the same dynamic patterns. The proposed procedure builds upon an existing framework [16], [17] which is based on the dynamics of social networks and the observation that communities generally remain stable. Here, we adapt and extend this methodology to account for dynamic datasets where the assumption of stability cannot be made, rendering the methodology more pertinent to the analysis of biological systems. We thus offer an additional solution procedure for dynamic community structure analysis, enabling a more in-depth insight into network organisation. Implementation as a Gephi plugin, named DyCoNet, allows the use of additional functionality through Gephi to analyse, visualise and enhance results. We demonstrate the applicability of the proposed methodology through relevant network examples below.

## Methods

### Approach

A dynamic network is a series of network snapshots at two or more time points, where time may represent seconds, hours, days etc. or multiple states of a system. Furthermore, as defined in [16], a *group* is a module in a partition of the network snapshot at time 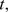 i.e. a group exists only at time 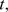 while a *community* is a dynamic module that persists across multiple snapshots. A two-stage approach for community structure detection in dynamic networks is described below.

**Figure 1:**
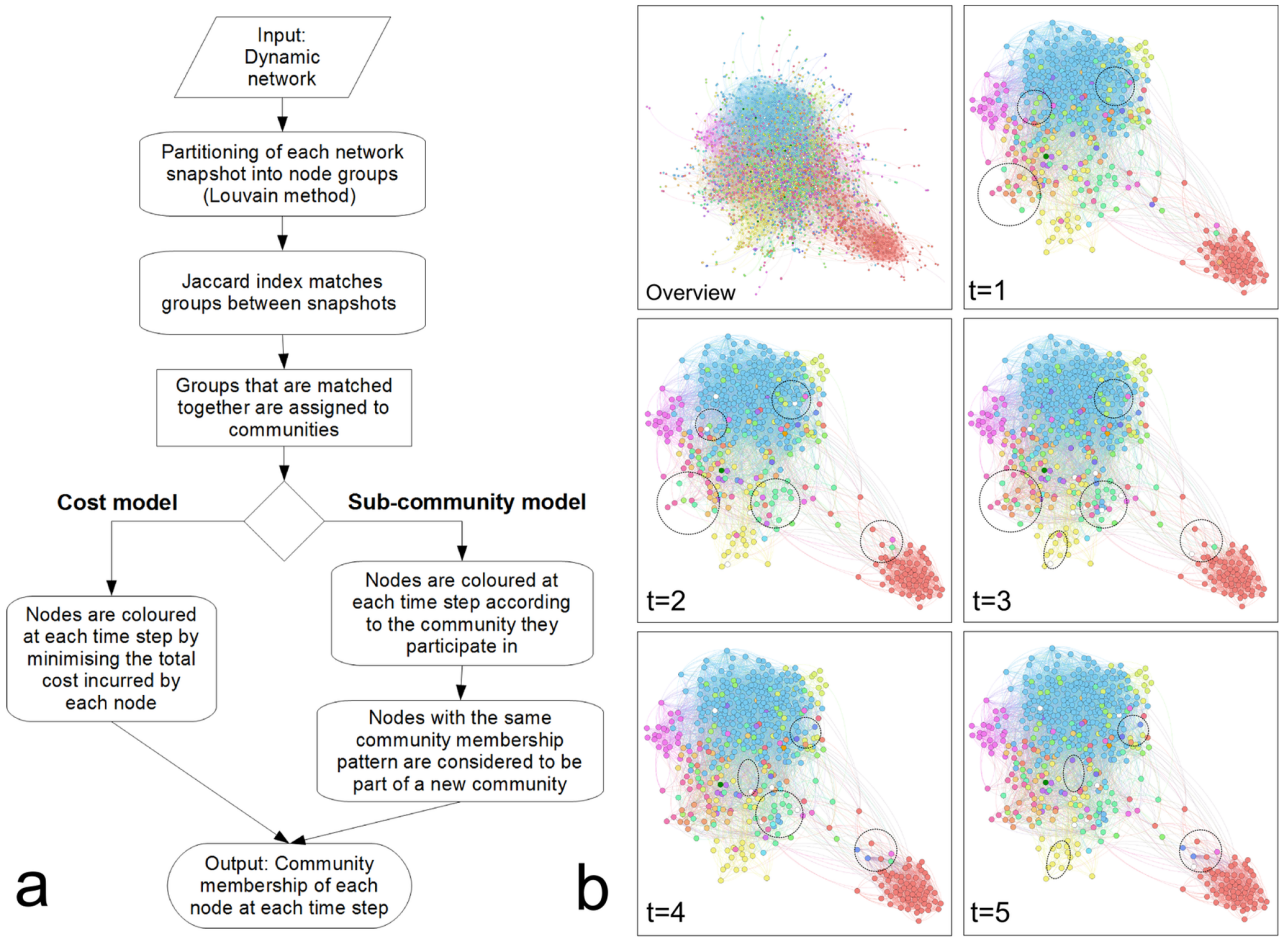
DyCoNet architecture and example analysis. a) Diagram depicting the analysis work-flow and plugin execution. b) Sub-community model applied on the TC-PIN from [14]. Top-left: An overview of the network, consisting of 3901 nodes and 16891 edges. Top-right to bottom-right: Snapshots of the network at time points 1–5. For clarity only the k-core of the network with 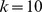 is shown. Node colours correspond to the community membership of each node. Layout of the network is done using the ForceAtlas2 algorithm. Parts of the network that change between time-points are indicated in circles to aid visualisation.

Stage one involves variations on elements of the methodology proposed in [16]. First, each network snapshot is partitioned independently into groups by the Louvain modularity optimisation method [18] and then each group at each time point is associated with a community. This was done previously by constructing a *group graph* and finding the minimum path cover to match groups across time points [16]. Here, for each group in the partition of the network snapshot at time point 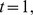 a community identifier is established. In other words, the groups that comprise the partition of the snapshot at 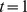 become initial reference communities. Instead of using the minimum path cover of the group graph, groups are now compared across time points using the Jaccard index (where a value of 1 indicates that two groups are identical) in order to assign a community identifier to each group. Next, each group at 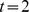 is compared with each group at 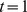 and assigned the community identifier of its most similar group. Subsequently, every group for 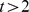 is compared with every other group at all previous time points, until a best match is found and the appropriate community identifier is assigned (Fig. 1a). A stopping criterion can limit how far back the algorithm will search for a good match. If there is no suitable match for a group, a new community is formed to which the group is associated. A user-defined parameter determines the value of the Jaccard index that constitutes an acceptable match, below which the formation of a new community takes place.

It should be noted that while groups pertain to specific time points and are independent of others, community structure is closely related to the time evolution of the network topology. The concept behind this is related to the dynamic nature of the network, where the networks are sampled in some natural order; the network partitions need to be aggregated meaningfully over time, so that the community structure corresponds to the time-series data. The communities are thus dependent on the order of the network partitions, since matches between snapshots in a different order would result in different community assignment. Therefore, the proposed community structure pertains to the specific time evolution of the data at hand and is not meaningful if the data is not analysed in the natural order over which it was sampled.

The first stage of the above procedure results in associating each node to a group and each group to a community for each time point. In the second stage, two different analytical procedures are considered to assign nodes to communities: the first is applicable to the scenario where communities are considered to be stable over time and the second, where this fact cannot be assumed. In the first instance, we adopt the *cost model* proposed previously by [16], to determine the community membership of each node at each time point. In this case, the model assumes that an individual tends not to change its ‘home’ community often, as based on observations from social networks, and a node can visit a community at time point 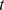 without being assigned to it. Communities are defined by matching across different time points as described previously. As community membership in this model is considered to be more or less stable, user-defined costs are incurred when a node *switches* community membership, *visits* another community of which it is not a member, or is *absent* from its community when other members of the community are present. Each node is assigned to a community at each time point by minimising the cost function via a dynamic programming algorithm [16]. This procedure identifies the loyalty of a node to its ‘home’ community and thus the stability of the community over time.

In the latter case, where the stability of communities cannot be assumed, we propose the *sub-community model*. Here, we offer an alternative solution procedure, which provides a deeper insight into the dynamic nature of community structure. In this model, a node is assigned the community to which its group is associated at each time point, without considering any incurred cost for its membership. In other words, we no longer consider the concept of a node visiting a community and generally staying loyal to a home community, but instead we accept that it is possible that a node may change communities often and therefore do not penalise it for doing so. This analysis leads to the identification of sets of nodes within communities (sub-communities) which exhibit highly variable community membership, but which all follow the same community membership pattern. For example, consider the case where nodes labelled 1, 2, 3, 4 and 5 in a dynamic network belong to community 1 at 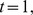 community 2 at 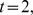 community 3 a 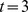 and finally community 1 again at 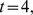 we define these nodes as displaying the same dynamic behaviour. Such groups of nodes with the same community membership pattern across various snapshots are considered to be part of a new sub-community (Fig. 1a).

### Implementation

The above approach is known as DyCoNet and has been implemented in the Java programming language using Gephi, an open-source software program used for graph visualisation and manipulation [19]. The Louvain algorithm which determines the groups at each time step is already available in Gephi. DyCoNet takes advantage of several classes from the Gephi library, particularly those found in the Attributes, Graph, Statistics, and Dynamic APIs. A workflow of the plugin execution is shown in Fig. 1a.

**Figure 2:**
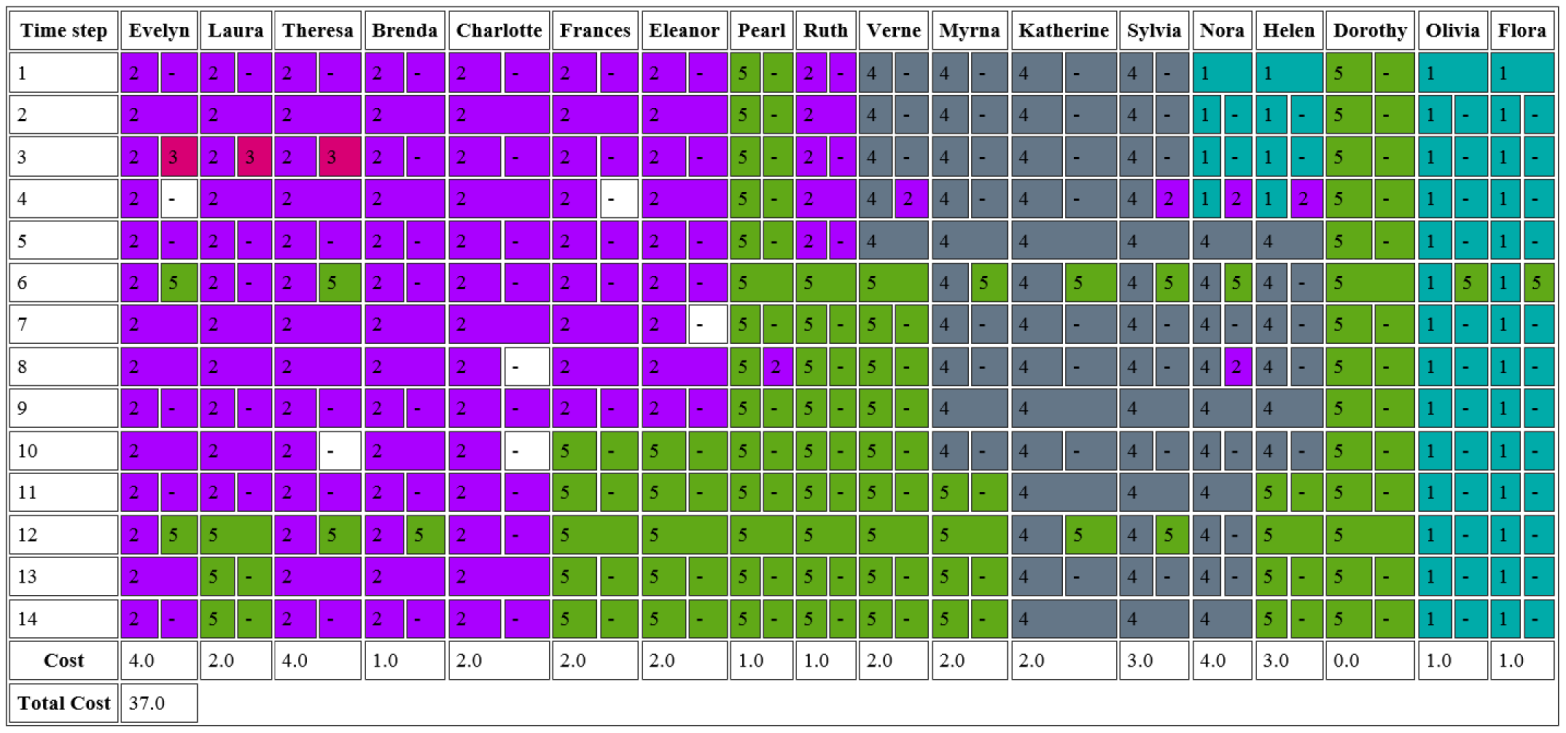
Communities found in the Southern Women dataset with Jaccard index group matching. For a detailed explanation of the output, please see text.

**Figure 3:**
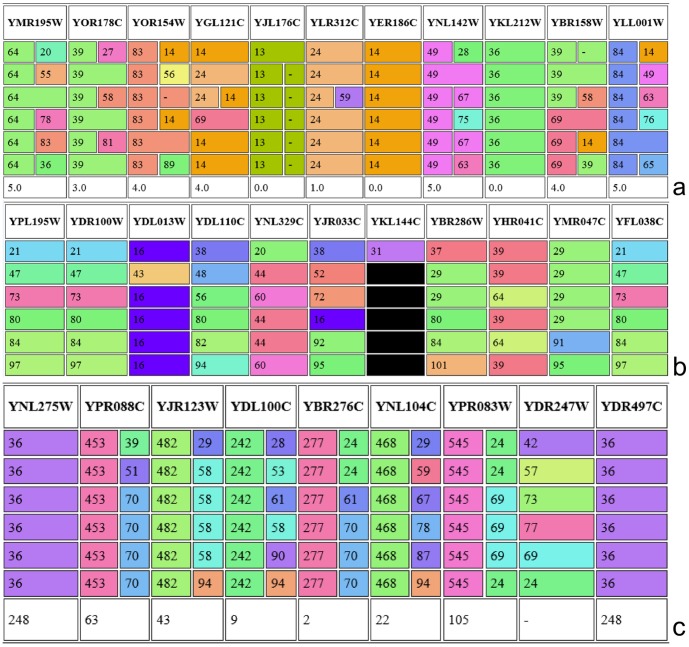
Community structure in the first 6 time steps on TC-PIN network from [14]. a) Identified communities using the social cost model, b) Assignment of proteins to the communities to which their groups are associated at each time point (i.e. no cost model), c) Identifying sub-communities of nodes with the same dynamic pattern. Colour and number of the first column indicate the sub-community that the protein has been assigned to, the second column the original community membership is shown. The number at the bottom of each column shows the number of members of the corresponding sub-community, or “-” if no sub-community has been assigned.

Gephi accepts network data from files in GEXF format. The user is presented with a tool-tip by hovering the cursor over an item to obtain a brief explanation of each feature. Before the plugin is executed the user selects which model to apply (cost or sub-community) and sets the parameter values. If the cost model is selected, the user must then set the visiting, switching and absence costs. In addition, the group matching behaviour can be modified by adjusting the Jaccard index and the search parameter, which control the similarity of the groups and how far back the algorithm will check for matching groups respectively. When the algorithm is executed, each node is assigned to its corresponding community. The colours of the nodes in the visual display of the network are then set to reflect the community membership of each node at each time step (Fig. 1b). The plugin outputs an HTML file containing a table with the community structure of the network at every time step (as shown in Fig. 2 and Fig. 3, discussed later), as well as a CSV file with the same information.

The analysis can be extended using other Gephi plugins. Using the built-in Timeline feature of Gephi, an animation of the evolution of the network structure can be viewed, illustrating how nodes change community over time. In addition, DyCoNet calculates the *node promiscuity*, which is the frequency with which a node changes its community affiliation. This can be represented visually using Gephi's ranking feature, where parameters such as the node size can be set to reflect the results generated by DyCoNet.

## Results and Discussion

The applicability of DyCoNet is demonstrated through two network examples. A relatively small social network that has been used previously and can serve as a motivating example to illustrate the features of the computational procedure is first described. Next, the application of DyCoNet to a dynamic protein interaction network is considered in more detail, in order to delineate the sub-community model in biological data where communities cannot be assumed to be stable.

### Small illustrative example

This example serves to illustrate how to use DyCoNet as well as how to interpret the output generated by the plugin. The Southern Women dataset was collected over a nine-month period by Davis et al. and followed the attendance of eighteen women at fourteen social events [16], [20]. The data set has been extensively studied and used as a benchmark for community identification methods [21]. The data used here was obtained from [16], where it is described as having stable communities thus making the dataset suitable for the cost model. We therefore apply DyCoNet to the Southern Women dataset, selecting the cost model analysis in stage 2 of the procedure and Fig. 2 shows the HTML report produced by this plugin.

The table in Fig. 2 depicts each community by a unique colour and corresponding number. The position 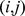 in the table indicates the community (or communities) associated with person 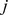 at time step 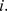 If two boxes with different colours are present at position 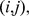 person 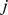 is visiting a community of which it is not a member. The box to the left of the cell indicates the person's ‘home’ community, and the box to the right indicates the community which they are visiting at that particular time step. If the right hand box contains a dash, then that person is absent. If the right-hand box contains a dash but has a colour, then both the community and the person are absent. In this case, an absence cost is not incurred since it is impossible for the person to be present when the community is also absent. If the box contains a dash but has no colour, then the person is absent but the community is present, thus incurring an absence cost. If the left-hand box of a cell is a different colour to the left-hand box directly above, then the person has switched communities.

Having discussed this simple example, we now move to a more complex biological network example to show how the method is adapted to deal with the less stable nature of the dataset.

### Networks with unstable community structure through a biological network example

DyCoNet was applied to a time-course protein interaction network (TC-PIN) constructed by mapping time series gene expression data onto static PPI networks [14] and the resulting dynamic network comprises a total of 3901 proteins over 6 time points. As mentioned previously, no assumptions of stability are applied here due the highly dynamic nature of molecular interactions. Therefore the sub-community model is deemed as the most appropriate option, however we first consider the application of the cost model to the TC-PIN to illustrate the difference in performance with the more stable social network dataset above.

The switching, visiting, and absence costs were set to the default to the default value of 1 (chosen according to [16]) and Fig. 3a shows part of the table generated by DyCoNet. Under the chosen cost parameters, the highest cost that can be incurred by a single node over 6 time steps is 5 and would correspond to a node that is a member of a different community at each time step. Half of the nodes shown in Fig. 3a incur a cost of 4 or 5, i.e. community membership is highly variable. To assess the stability of the communities we calculated the average cost for each node per time step, equal to 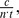 where 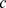 is the total cost, 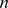 the number of nodes and 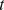 the number of time steps. In the previous example, assuming a total cost of 37 found by DyCoNet (Fig. 2a), the average cost is 0.147. For the TC-PIN example, the corresponding cost is 0.5. Thus the change in community per node per time step is considerably higher for the TC-PIN network, reflecting the greater frequency by which nodes in the biological network example change their community membership, therefore rendering the cost model less suitable.

This effect can be further illustrated by the protein YMR195W (first column of Figure 3a). YMR195W participates in a different community at each time step but is assigned community 64 as its home community for all time steps by the cost model. On inspection, it does not seem reasonable to consider protein YMR195W to be a permanent member of community 64, when it only participates in that community at one time step. Similarly, the protein YOR154W (column 3) is assigned to community 83 at each time step, although only actively participating in the community at one time step. Therefore, this variable community membership is taken into consideration and the sub-community model is now applied to the dataset.

As described in the Approach section, each protein now belongs to the community to which its group is associated at each time point (Fig. 3b). The sub-community model searches for sets of nodes which follow the same community membership pattern and creates new sub-communities with the same dynamic behaviour. For example, in Fig. 3b, proteins YPL195W, YDR100W and YFL038C change communities several times, but at each time step all three proteins belong to the same community, therefore these proteins would form a new sub-community.

The output generated by our method is illustrated in Fig. 3c, where a portion of the entire output table generated by the sub-community model is shown. The first column indicates the sub-community membership of each protein, while the second column shows the community it was originally assigned with the Jaccard index matching. For nodes where one column is present, either: (i) the protein stays in the same community throughout and thus its sub-community is identical to its original community, or (ii) the pattern of community membership for this node is unique and so it is not assigned to any sub-community, indicated by a dash in the last row of that column. The number at the bottom of each column indicates the number of nodes that belong to the corresponding community or sub-community.

More specifically, in Fig. 3c the first column indicates that protein YNL275W is a member of community 36 at each time step and there are 248 such proteins, including protein YDR497C in the last column of the table. The second column indicates that protein YPR088C participates in community 39 at time step 1, in community 51 at time step 2, and in community 70 for the remaining 4 time steps. 63 other proteins follow this dynamic pattern and the sub-community comprising these proteins is labelled 453. As can be seen in the penultimate column, protein YDR247W changes communities at each time step and does not follow the same pattern as any other protein in the network, therefore no sub-community label is assigned. Overall, 16 communities are found in the first stage by mapping groups based on the Jaccard index and 480 sub-communities are found in stage 2 of the procedure.

**Figure 4:**
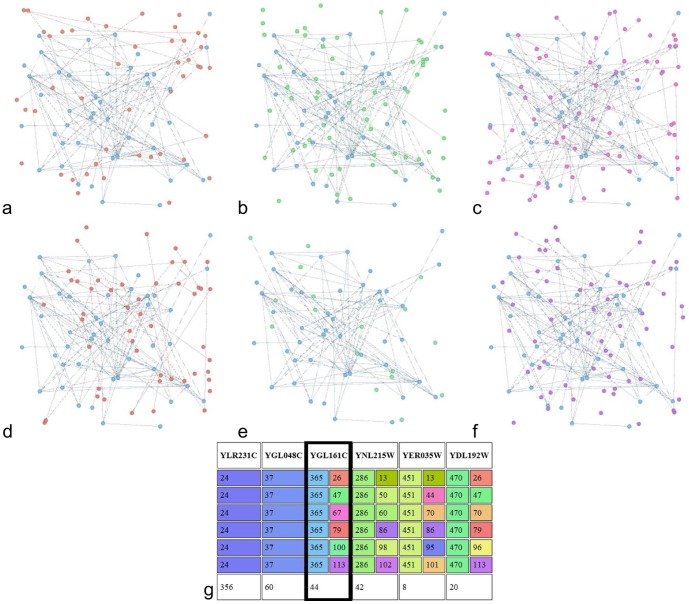
Sample sub-community for all 6 time steps. a-f) Cyan coloured nodes correspond to the sub-community discovered from the set of nodes with the same community change pattern. Rest of coloured nodes are the ones belonging in the original communities, with the original colour shown. g) A sample protein belonging to the depicted community (left column) and its corresponding community pattern change (right column).

Figure 4 shows the changing community membership of nodes from a sample sub-community. These nodes (cyan coloured) change communities across time but follow the same community membership pattern at each time point, and therefore become a sub-community. The remaining nodes of the corresponding community at each time point are also shown. Thus, the sub-community nodes remain the same colour for all time steps, while the colour of the surrounding nodes changes. It is obvious that a significant number of nodes behaves similarly, changing communities across time points with the same pattern, which justifies the application of the sub-community model in cases where the stability of communities cannot be assumed. Overall, out of 3901 proteins in our dataset, the vast majority (2400) were assigned to 480 sub-communities detected by DyCoNet.

**Figure 5:**
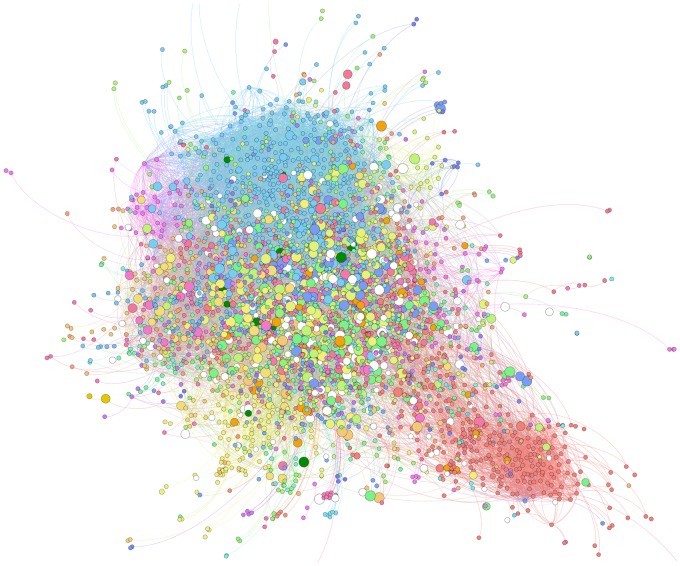
TC-PIN for all 6 time steps from [14]. Node colours correspond to sub-communities identified by DyCoNet, node size corresponds to node promiscuity i.e. frequency with which nodes change communities. This visualisation is produced in Gephi by setting the ranking feature of each node such that ‘Node Promiscuity’ corresponds to node size.

An important aspect of our method is the capability to visualise the dynamic behaviour of the community structure for any given input network. Fig. 5 shows the final community/sub-community membership found by the sub-community model across 6 time steps of the TC-PIN network described above. A node either belongs to a sub-community or a community, if it was not assigned a sub-community in part 2 (e.g. YNL275W and YDR497C in Fig. 3c). The stability of node-community membership is visualised by setting node size proportional to node promiscuity. As seen in the figure, there are far fewer proteins that frequently change communities (large nodes) compared to the ones that don't (small nodes), indicating a stable community structure (i.e. many nodes with the same dynamic community pattern), when considering the sub-community model. Therefore, in cases such as this where nodes frequently change communities, the sub-community model may produce a more realistic community structure representation by identifying the most stable sets of nodes.

We have also investigated the effect of changing the value of the Jaccard index (default value equal to 0.4) that constitutes an acceptable match between groups to form communities (Table S1). As this threshold value increases, the average size of the communities identified decreases dramatically. This is because as the matching criterion becomes more strict, it is increasingly difficult to match the groups between the time-points and form communities. The average size of the sub-communities identified, however, increases slowly, with no noticeable trend in the number of sub-communities. With roughly 500 sub-communities identified for various Jaccard index cut-off values, it seems that the number of sub-communities is not significantly affected and thus the community structure is relatively stable. As the Jaccard index threshold increases, so does the average node promiscuity, with unstable nodes changing communities more frequently as groups are matched with more stringent criteria.

## Conclusions

We have implemented and extended a community structure detection method derived from the analysis of social networks. Our extension renders the method more suitable to networks where no assumptions on stability can be made, making it particularly suitable for biological data, which can be highly variable or even noisy. The proposed procedure is implemented as a Gephi plugin, known as DyCoNet, that is available for download.

The software implementation is flexible and easy to use. DyCoNet offers two solution procedures, whose suitability is dependent on the nature of the dataset being studied. Furthermore, two user-defined parameters, i.e. the Jaccard search and cutoff parameters allow the analysis to be tailored to different experimental needs. The use of additional Gephi functionality allows for enhanced analysis and visualisation of results. Network representations of results can emphasize node characteristics such as rate of change of community membership (i.e. node promiscuity) through the ranking parameter facility (Fig. 5). Furthermore, an animation of the community structure across different time points can be visualised using the built-in time-line feature. DyCoNet also produces results in table and CSV format to facilitate further processing.

We have demonstrated the applicability of DyCoNet on two network examples with widely different dynamic behaviour and showed that the method is well-adapted to both cases. Therefore, DyCoNet can be applied to networks with a variety of topological characteristics across different disciplines. This software can pave the way for novel applications in dynamic networks, so as to facilitate a deeper insight into the underlying dynamic organisation of the target system.

## Supporting Information

Table S1 **Effect of the value of Jaccard index used in group matching on community size, number and node promiscuity.**
(PDF)
