# Supplementary material for: DyCoNet: A Gephi Plugin for Community Detection in Dynamic Complex Networks
Source: PLoS One. 2014 Jul 7;9(7):e101357. doi: 10.1371/journal.pone.0101357 (PMC4084810; doi:10.1371/journal.pone.0101357)
Supplement: Table S1 — Effect of the value of Jaccard index used in group matching on community size, number and node promiscuity. (PDF) [file pone.0101357.s001.pdf]

DyCoNet: a Gephi Plugin for community detection in dynamic biological networks: Supporting Information

Julie Kauffman[1], Aristotelis Kittas[1], Laura Bennett[2], Sophia Tsoka[1,*]
1 Department of Informatics, King's College London, Strand, London WC2R 2LS, United Kingdom
2 Centre for Process Systems Engineering, Department of Chemical Engineering, University College London, Torrington Place, London WC1E 7JE, United Kingdom
∗ **E-mail: sophia.tsoka@kcl.ac.uk**

Tables

**Jaccard Index**	**0.4**	**0.5**	**0.6**	**0.7**	**0.8**	**0.9**
**Number of communities identified**	15	15	14	14	13	13
**Number of sub-communities identified**	500	482	498	465	478	504
**Average community size**	42.3	42.3	19.4	19.8	2	2
**Average sub-community size**	4.8	5.17	5.6	6.1	6.39	6.03
**Average node promiscuity**	1.74	1.73	1.83	1.85	1.9	1.94

Table S1. Effect of the value of Jaccard index used in group matching on community size, number and node promiscuity.
